# Modulation of Insulin Resistance and the Adipocyte-Skeletal Muscle Cell Cross-Talk by LCn-3PUFA

**DOI:** 10.3390/ijms19092778

**Published:** 2018-09-15

**Authors:** Alexandre Pinel, Jean-Paul Rigaudière, Chrystèle Jouve, Frédéric Capel

**Affiliations:** Unité de Nutrition Humaine (UNH), INRA/Université Clermont Auvergne, 63009 Clermont-Ferrand, France; alexandre.pinel@uca.fr (A.P.); jean-paul.rigaudiere@inra.fr (J.-P.R.); christelle.jouve@inra.fr (C.J.)

**Keywords:** insulin resistance, omega-3 fatty acids, adipocytes, myotubes, conditioned media

## Abstract

The cross-talk between skeletal muscle and adipose tissue is involved in the development of insulin resistance (IR) in skeletal muscle, leading to the decrease in the anabolic effect of insulin. We investigated if the long chain polyunsaturated n-3 fatty acids (LCn-3PUFA), eicosapentaenoic and docosapentaenoic acids (EPA and DPA, respectively) could (1) regulate the development of IR in 3T3-L1 adipocytes and C2C12 muscle cells and (2) inhibit IR in muscle cells exposed to conditioned media (CM) from insulin-resistant adipocytes. Chronic insulin (CI) treatment of adipocytes and palmitic acid (PAL) exposure of myotubes were used to induce IR in the presence, or not, of LCn-3PUFA. EPA (50 µM) and DPA (10 µM) improved PAL-induced IR in myotubes, but had only a partial effect in adipocytes. CM from adipocytes exposed to CI induced IR in C2C12 myotubes. Although DPA increased the mRNA levels of genes involved in fatty acid (FA) beta-oxidation and insulin signaling in adipocytes, it was not sufficient to reduce the secretion of inflammatory cytokines and prevent the induction of IR in myotubes exposed to adipocyte’s CM. Treatment with DPA was able to increase the release of adiponectin by adipocytes into CM. In conclusion, DPA is able to protect myotubes from PAL-induced IR, but not from IR induced by CM from adipocytes.

## 1. Introduction

In 2060, the European population is expected to reach 517 million and one third of people will be more than 65 years old [1]. This represents a significant public health challenge with a major economic cost, notably because of several associated chronic diseases. Variations in lean and fat masses have common pathophysiological mechanisms, including insulin-resistance (IR), a low-grade inflammation [2], and specific dysfunctions of adipose tissue and skeletal muscle metabolisms. Altogether, these abnormalities are involved in the development of anabolic resistance of skeletal muscle cells and the progressive muscle atrophy observed during aging.

It has been well described that obesity induces changes in the secretory activity of adipose tissue. Chronic inflammation of adipose tissue decreases adiponectin (an adipokine with insulin-sensitizing and anti-inflammatory effects) production whereas the release of pro-inflammatory cytokines (interleukine-6 (IL-6), monocyte chemoattractant protein-1 (MCP-1), and tumor necrosis factor alpha (TNF-α) is increased [3]. The contribution of IL-6 and TNF-α production by muscle cells [4] to systemic inflammation is unclear but it was shown that chronically increased plasma levels of IL-6 and TNF-α could mediate insulin and anabolic resistance of skeletal muscle’s cells [5,6].

In addition, combination of a high energy intake and insulin resistance (IR) in adipose tissue increase the net efflux of free fatty acids (FA) from adipose tissue into the blood circulation [7]. Free FA will be caught up by the liver and skeletal muscles, leading to ectopic fat deposition. In insulin-sensitive tissues, the increase of intracellular triglycerides (TG) is associated with the accumulation of lipid intermediates, such as diglycerides (DG) and ceramides. These molecules can activate protein kinase C (PKC) [8,9], which inhibits protein kinase B (PKB, also known as Akt), a crucial effector of insulin signaling. Therefore, these lipotoxic events could participate in the development of resistance to the anabolic action of insulin and the loss of skeletal muscle’s function.

Nutritional strategies are currently developed to prevent IR and metabolic disturbances. Recently, we have shown that long-chain omega-3 polyunsaturated fatty acids (LCn-3PUFA) could protect skeletal muscle cells from IR. Eicosapentaenoic acid (EPA, 20:5n-3) and docosahexaenoic acid (DHA, 22:6n-3) supplementations improved Akt activation and glucose uptake in vitro by the inhibition of PKC activation and the accumulation of intracellular DG and ceramides that are induced by an exposure to palmitic acid (PAL) [10]. Furthermore, it has also been shown that DHA could improve the growth of myotubes in a model of PAL-induced atrophy [11].

Among the different LCn-3PUFA found in human tissues, less attention was given to docosapentaenoic acid (DPA, 22:5n-3) which is produced during the conversion of EPA into DHA. Nevertheless, some evidences showed that DPA was as efficient as EPA and DHA in the prevention of metabolic disorders. Its plasma level was associated with fewer coronary heart disease deaths [12]. DPA could also inhibit cyclooxygenase and the production of pro-inflammatory prostaglandins by macrophages [13]. Interestingly, we recently observed in mice fed with a high fat, high sucrose diet supplemented with EPA an increase in DPA content in different tissues concomitantly to an improvement in insulin sensitivity as compared to non-supplemented animals [14].

LCn-3PUFA are quite prone to beta-oxidation as well as incorporation into phospholipids (PL). It could be then hypothesized that their effects are, at least partially, mediated by the modulation of the membrane’s PL composition. Indeed, the FA composition of PL regulates the fluidity of cytoplasm or intracellular membranes and could be involved in insulin signaling efficiency [15].

Considering all these elements, the present study aimed at evaluating the effect of DPA on the interrelation between adipose and muscle cells in the context of IR. Our objectives were (1) to explore the reversion of IR by EPA and DPA in adipocytes and muscle cells; (2) to determine the impact of adipocyte IR on the induction of muscle IR; and (3) to evaluate the potential mechanisms and mediators involved in the cross-talk between adipocytes and myotubes.

## 2. Results

### 2.1. Effects of LCn-3PUFA Treatments on Membrane Phosphatidylcholine Content and Fatty Acid Profile

**Figure 1:**
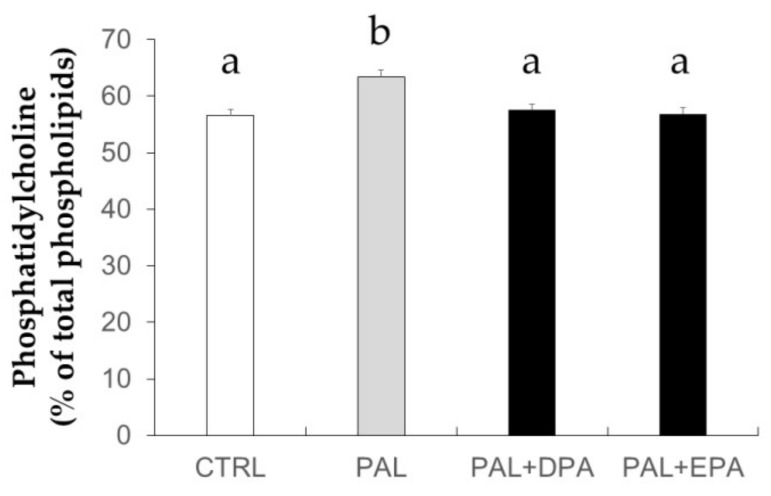
Phosphatidylcholine content in membranes of C2C12 muscle cells. C2C12 muscle cells were treated with 500 µM palmitic acid (PAL) with or without 10 µM docosapentaenoic acid (DPA) or 50 µM eicosapentaenoic acid (EPA). Cells were harvested for lipid extraction and phospholipids were separated by high performance liquid chromatography (HPLC). Phospholipid classes were quantified and expressed as the percent of total phospholipids. Data are mean ± SEM (*n* = 4–6 obtained after three experiments). Different letters mean significant differences (*p* < 0.05) between groups obtained by ANOVA and the Tukey post-hoc test.

In order to validate the effectiveness of LCn-3PUFA treatments on the modification of the profile of membrane’s PL, the FA composition of phosphatidylcholine (PC) has been evaluated (Table 1). In 3T3-L1 adipocytes, the amount of PC was not significantly affected by CI or FA treatment (data not shown). DPA relative abundance in PC was enhanced in adipocytes exposed to DPA compared to those treated with chronic insulin (CI) only (*p* = 0.013 vs. CI). Similarly, EPA treatment significantly increased EPA relative abundance in PC compared to CI condition alone (*p* < 0.0001 vs. CI). In C2C12 muscle cells treated directly with FA, DPA and EPA also exhibited a significant incorporation into PC compared to cells treated with PAL alone. As shown in Figure 1, treatment with PAL significantly increased the PC relative amount compared to control (CTRL) (+ 6%, *p* = 0.005, PAL vs. CTRL). Treatment with LCn-3PUFA restored the PC relative amount to the CTRL value.

### 2.2. Effect of Fatty Acid Treatments on Insulin Resistance in C2C12 Muscle Cells

**Figure 2:**
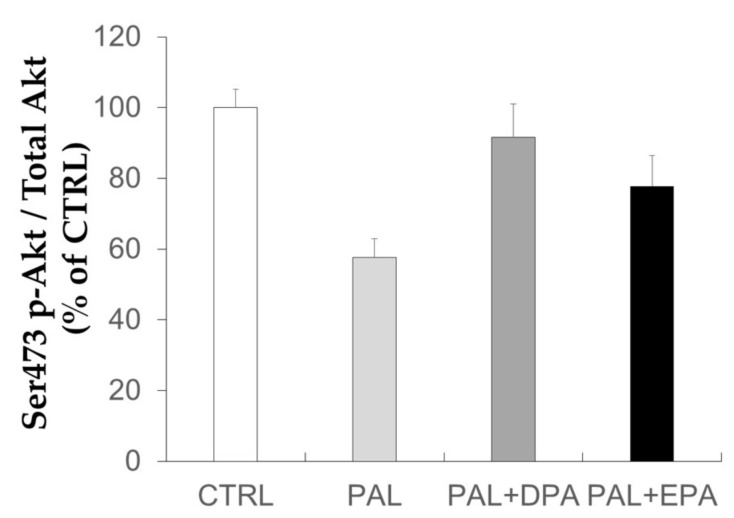
Effects of palmitic acid and LCn-3PUFA on insulin signaling in skeletal muscle cells. C2C12 muscle cells were treated with 500 µM palmitic acid (PAL) with or without 10 µM docosapentaenoic acid (DPA) or 50 µM eicosapentaenoic acid (EPA). Before harvesting, cells were stimulated with insulin (100 nM) to evaluate insulin sensitivity by the quantification of Akt protein phosphorylation. Data are mean ± SEM (*n* = 11–14 obtained after six experiments). Different letters mean significant differences (*p* < 0.05) between groups obtained by ANOVA and the Tukey post-hoc test.

As PAL can induce IR in skeletal muscle cells, we investigated the effects of DPA on PAL-induced IR in C2C12 muscle cells (Figure 2). Akt protein is almost not phosphorylated in the absence of insulin stimulation and FA alone have no significant effect (Figure S1). The effect of FA was then investigated in cells exposed to insulin for 10 min. PAL treatment for 16 h significantly decreased Akt ser473/4 phosphorylation after insulin stimulation. Similar observations could be observed on thr308/9 residue [10]. When medium was supplemented with LCn-3PUFA, Akt phosphorylation tended to be improved by EPA (*p* = 0.18, EPA vs. PAL) and was restored by DPA (*p* = 0.015, DPA vs. PAL).

### 2.3. Chronic Insulin Induced Insulin Resistance in 3T3-L1 Adipocytes

**Figure 3:**
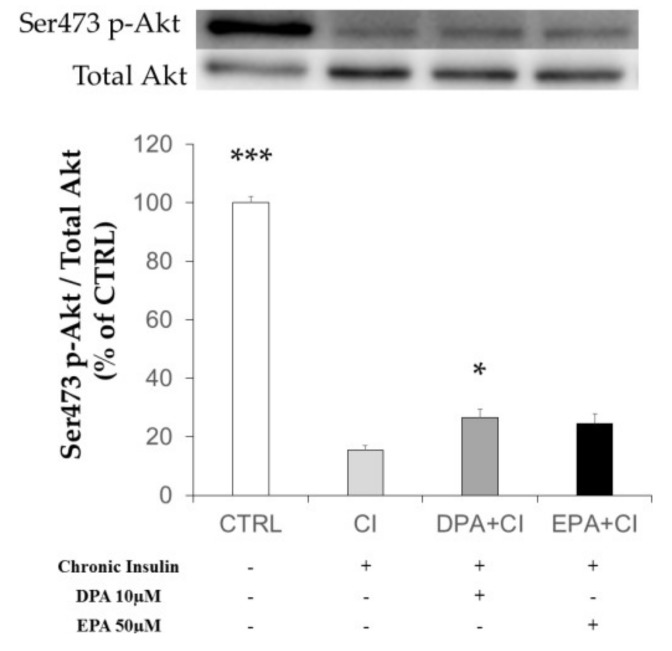
Effects of chronic insulin and LCn-3PUFA on insulin signaling in mature adipocytes. 3T3-L1 preadipocytes were differentiated for eight days before a 48 hour-treatment with 10 µM docosapentaenoic acid (DPA) or 50 µM eicosapentaenoic acid (EPA). Insulin resistance was induced by adding insulin (10 µM) during the last 16 h of treatment (chronic insulin, CI). Cells were then starved for 6 h. Before harvesting, cells were stimulated with insulin (100 nM) to evaluate insulin sensitivity by the quantification of Akt protein phosphorylation. Data are mean ± SEM (*n* = 10 in CI and control (CTRL) groups and *n* = 20 in LCn-3PUFA groups obtained after four experiments.) * *p* < 0.05 vs. CI and *** *p* < 0.001 vs. CI were obtained by ANOVA followed by the Dunnett post-hoc test.

We investigated the effect of chronic insulin on 3T3-L1 adipocytes previously exposed to LCn-3PUFA or not (Figure 3). It has been previously shown that serum starved adipocytes exhibited a very poor basal Akt protein activation [16]. We then exposed 3T3-L1 adipocytes to insulin for 10 min after serum and insulin deprivation to explore the phosphorylation of Akt protein as an index of insulin sensitivity. We observed that CI treatment induced a strong decrease in insulin-dependent Akt phosphorylation on ser473/4 residue (*p* < 0.001 vs. CTRL). Although the improvement did not restore Akt activation to the control value, pre-incubation of adipocytes with EPA tended to partially restore, while DPA partially and significantly restored, Akt phosphorylation (*p* = 0.1 and *p* = 0.031 vs. CI condition, respectively).

### 2.4. Effects of Chronic Insulin and LCn-3PUFA Treatments on Gene Expression in 3T3-L1 Adipocytes

**Figure 4:**
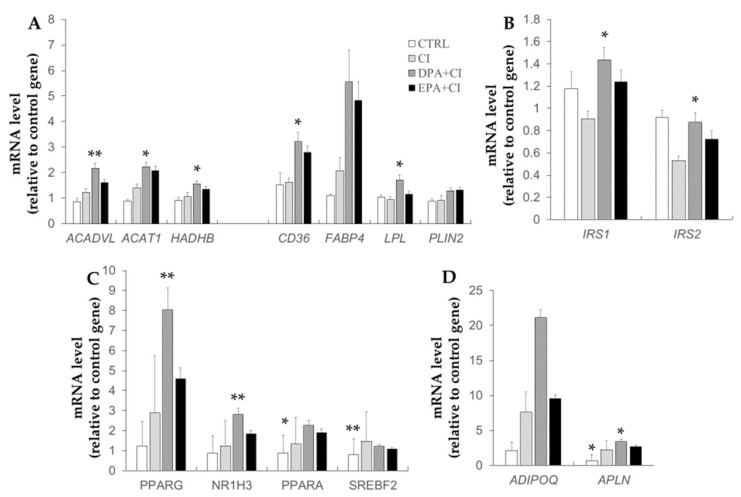
Effects of chronic insulin and LCn-3PUFA on gene expression in 3T3 adipocytes. 3T3-L1 preadipocytes were differentiated for eight days before a 48-hour treatment with 10 µM docosapentaenoic acid (DPA) or 50 µM eicosapentaenoic acid (EPA). Insulin resistance was induced by adding chronic insulin (CI, 10 µM) during the last 16 h of treatment. Cells were then starved for 6 h and RNA were collected to perform qPCR. Relative gene expression of genes related to lipid metabolism (**A**), insulin signaling (**B**), transcription factors (**C**), and regulators of energy metabolism (**D**) are presented. The *NONO* gene was used as the housekeeping gene. Data represent the mean ± SEM (n = 5–6 obtained after three experiments). * *p* < 0.05; and ** *p* < 0.01 vs. CI obtained by ANOVA followed by the Dunnet post-hoc test.

The mRNA levels of genes related to lipid metabolism, insulin signaling, encoding key transcription factors involved in lipid or FA metabolisms and regulators of energy metabolism were evaluated in 3T3-L1 adipocytes exposed to CI and LCn-3PUFA (Figure 4). The mRNA amount of genes encoding for proteins involved in lipid mitochondrial oxidation (*ACADVL, ACAT1, HADHB*) were significantly enhanced by DPA (*p* = 0.004, *p* = 0.02 and *p* = 0.032 vs. CI, respectively) (Figure 4A). EPA tended to increase *ACAT1* mRNA expression (*p* = 0.055 vs. CI). The mRNA level of *CD36* was enhanced by DPA and EPA, but only cells treated with DPA exhibited a significant difference with the CI group (*p* < 0.05 vs. CI and *p* = 0.071 vs. CI, respectively). *FABP4* and *LPL* mRNA expression was enhanced by DPA treatment compared to CI group (*p* = 0.078 and *p* = 0.014 vs. CI, respectively). *PLIN2* mRNA expression was also slightly enhanced by DPA and EPA treatments (*p* = 0.109 and *p* = 0.065 vs. CI, respectively). As shown in Figure 4B, *IRS2* mRNA level strongly tended to be reduced in CI group vs. CTRL group (*p* = 0.062). Supplementation for 48 h with DPA significantly enhanced both *IRS1* and *IRS2* gene expression compared with CI alone (*p* = 0.019 and *p* = 0.034 vs. CI, respectively). *PPARG*, *NR1H3* (coding for LXRα1), and *PPARA* mRNA expressions were all enhanced by DPA supplementation compared to CI treatment alone (*p* = 0.002, *p* = 0.003 and *p* = 0.035 vs. CI, respectively) (Figure 4C). CTRL and EPA+CI groups exhibited a lower expression of *SREBF2* mRNA compared to CI (*p* = 0.004 and *p* = 0.06 vs. CI, respectively). No effect of DPA supplementation was observed on *SREBF2* gene expression. *ADIPOQ* (adiponectin) gene expression strongly tended to be enhanced by DPA supplementation vs CI treatment alone (*p* = 0.078 vs. CI) (Figure 4D). Finally, *APLN* gene expression was reduced in the CTRL group (*p* = 0.02 vs. CI), whereas mRNA level was increased by DPA treatment compared to CI (*p* = 0.046 vs. CI). EPA treatment had no effect on *APLN* gene expression compared to CI.

### 2.5. Effects of Chronic Insulin and LCn-3PUFA Treatments on Mature Adipocyte Secretion

**Figure 5:**
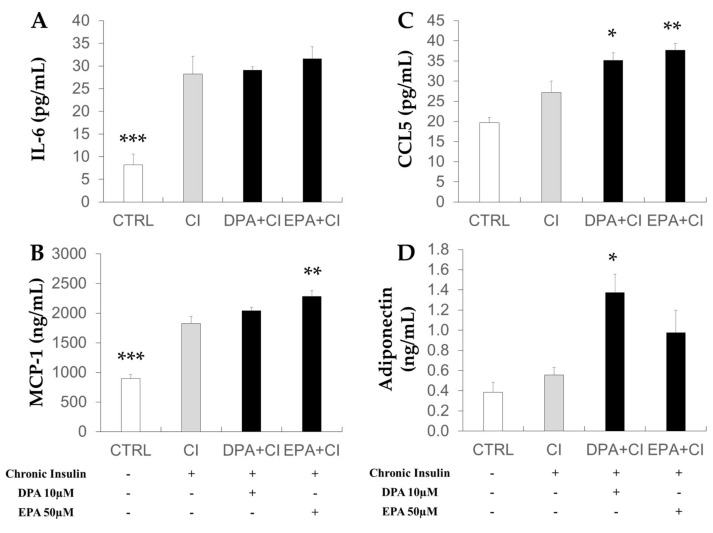
Effects of chronic insulin and LCn-3PUFA on adipokine secretion by mature adipocytes. 3T3-L1 preadipocytes were differentiated for eight days before a 48-hour treatment with 10 µM docosapentaenoic acid (DPA) or 50 µM eicosapentaenoic acid (EPA). Insulin resistance was induced by adding insulin (10 µM) during the last 16 h of treatment (chronic insulin, CI). Cells were then starved for 6 h and conditioned media were collected for quantification of IL-6 (**A**), MCP-1 (**B**), CCL5 (**C**), and adiponectin (**D**) concentrations. Data represent the mean ± SEM (*n* = 8–12 obtained after three experiments). * *p* < 0.05; ** *p* < 0.01; and *** *p* < 0.001 vs. CI obtained by ANOVA followed by the Dunnet post-hoc test. CCL-5: C-C motif chemokine ligand 5; IL-6: interleukin-6; MCP-1: monocyte chemoattractant protein-1.

Adipokine and chemokine secretions were analyzed in conditioned media (CM) collected after starvation of adipocytes for 6 h (Figure 5). As compared to CI treatment, IL-6 secretion was lower in control adipocytes compared to insulin-resistant adipocytes (CI). EPA and DPA treatment of CI adipocytes had no effect (Figure 5A). MCP-1 secretion was increased by EPA supplementation (*p* < 0.01 vs. CI) and C-C motif chemokine ligand 5 (CCL5) secretion was increased by DPA and EPA supplementation compared to CI condition (Figure 5B,C). Adiponectin secretion (Figure 5D) was increased by DPA supplementation (*p* < 0.05 vs. CI).

### 2.6. Effect of Adipocyte-Conditioned Media on Akt Phosphorylation in C2C12 Muscle Cells

**Figure 6:**
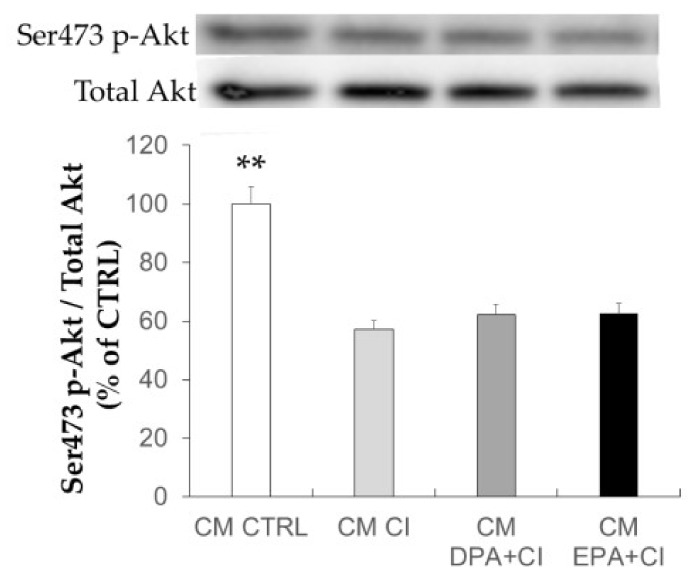
Effects of adipocyte condition media on Akt activation in skeletal muscle cells. Adipocyte media were collected after 48 h of treatment with, or without, 16-hour chronic insulin (CI), docosapentaenoic acid (DPA), oreicosapentaenoic acid (EPA), and after six hours of starvation. C2C12 muscle cells were treated for 16 h with conditioned media (CM). Before harvesting, cells were stimulated with insulin (100 nM) to evaluate insulin sensitivity by the quantification of Akt protein phosphorylation. Data are represented as the mean ± SEM (*n* = 10 in CI and control (CTRL) groups, and *n* = 20 in LCn-3PUFA groups obtained after four experiments. ** *p* < 0.01 vs. CI obtained by ANOVA followed by the Dunnet post-hoc test.

CM collected after starvation for 6 h did not contain FA (data not shown) or insulin allowing the characterization of the effects of adipocyte secretions. In C2C12, CM from insulin-resistant adipocytes significantly decreased the response to insulin as demonstrated by the lower Akt phosphorylation on ser473/4 residue compared to control C2C12 (*p* < 0.01 vs. CTRL) (Figure 6). CM from adipocyte supplemented with LCn-3PUFA also induced IR as well, without any preventive effect.

## 3. Discussion

Several studies have previously demonstrated the beneficial effects of EPA or DHA, two LCn-3PUFA on IR in skeletal muscle cells exposed to high concentrations of PAL [10,17,18]. The alteration in the activation of Akt could be considered as a first step in the development of IR. We previously showed that LCn-3PUFA can normalize Akt activation by insulin on both serine and threonine residues in C2C12 myotubes exposed to PAL [10]. DPA, an intermediate of DHA production from EPA, remains poorly studied. Here we showed that DPA which is less abundant in plasma membranes than EPA or DHA, is as efficient as other LCn-3PUFA to improve muscle insulin sensitivity in vitro, even when used at a lower concentration (10 µM compared to 30–50 µM of EPA or DHA). This effect is due to a reversion of the lipotoxic effect of PAL. Hence, LCn-3PUFA without PAL, are not able to enhance the effect of insulin on Akt phosphorylation (Figure S1). The enrichment of membrane’s PL with LCn-3PUFA could facilitate protein interactions in cellular membranes and then play a role in the modulation of insulin signaling [15]. A net increase of DPA and EPA concentration was observed in PC from both muscle and adipose cells after 16 to 48 h of incubation. It might partially explain the beneficial effects of LCn-3PUFA. In adipocytes, only a slight improvement of insulin-induced Akt activation was observed when cells were supplemented with LCn-3PUFA before the induction of IR. The model of insulin-resistant adipocytes was first described by Kozka et al. in 1991 [19] who demonstrated a decrease of cell surface glucose transporter 4 in 3T3-L1 adipocytes after chronic exposure to insulin. To further explore the effect of LCn-3PUFA on the cellular effects of insulin downstream of Akt, the exploration of Akt substrate of 160 kDa (AS160) activation, and glucose transporter 4 translocation to plasma membrane will be of particular interest.

In our study, LCn-3PUFA only partially improved IR induced by CI in adipocytes but induced some metabolic adaptations, especially on lipid metabolism. We observed an increase of FAT/CD36 expression with DPA and EPA, DPA having a stronger effect. FAT/CD36 can also modulate insulin sensitivity and the incorporation of essential FA in 3T3-L1 adipocytes [20]. DPA significantly enhanced the expression of lipid mitochondrial oxidation markers (*ACAT1, ACADVL, HADHB*). This is in perfect agreement with previous results published by Madsen et al. showing that the mitochondrial oxidation of FA was enhanced by EPA and DHA in fully differentiated 3T3-L1 adipocytes [21]. DPA also induced some changes in the expression level of genes involved in insulin signaling. Expression of *IRS1* and *IRS2* mRNA was enhanced in DPA-treated adipocytes. IRS1 and IRS2 were identified as crucial regulators of adipocyte differentiation as simultaneous knock out of these genes completely blocked differentiation in mice [22]. In our study, we incubated fully-differentiated adipocytes with LCn-3PUFA. We could then hypothesize that DPA may affect the maintenance of these cells, suggesting a preserved capacity for FA and glucose buffering during IR and with over-nutrition. Supporting this idea, the mRNA levels of *PPARG*, *PPARA*, and *NR1H3*, which are crucial transcription factors involved in differentiation and lipid metabolism, were increased in adipocytes exposed to DPA. The exact time-window of the treatment of adipocytes with LCn-3PUFA might be critical as it has been shown that only DHA treatment during the differentiation process had inhibitory effects [23], whereas EPA or DHA incubation before and during the induction of differentiation increased adipogenesis [24].

Although we found marked changes in the mRNA level of key metabolic genes following DPA treatment, it was not sufficient to reduce the ability of adipocyte-CM to induce IR in skeletal muscle cells. Induction of IR in mature adipocytes resulted in a higher secretion of adipokines involved in inflammation and macrophage recruitment (MCP-1 and CCL5). These increases were not reversed by treatment with LCn-3PUFA. Surprisingly, DPA and EPA further increased CCL5 secretion and EPA also had an enhancing effect on MCP-1 secretion by adipocytes. MCP-1, CCL5 (also known as regulated upon activation normal T cell expressed and secreted, RANTES) and IL-6 secretions are known to be enhanced in mature adipocytes compared to undifferentiated adipocytes [25,26]. Nevertheless, incubation of muscle cells with IL-6 for less than 96 h is known to have an insulin sensitizing effect [27], whereas MCP-1 is able to impair insulin signaling [28]. DPA also significantly improved adiponectin secretion and tended to increase its gene expression. Adiponectin has anti-inflammatory and insulin-sensitizing properties [29], which could then have a protective role against metabolic abnormalities in other tissues. However, the beneficial effects of this adipokine could be masked by an opposite action of pro-inflammatory chemokines secreted by adipocytes.

The factors involved in the induction of IR from adipocytes to skeletal muscle cells remains unknown. Literature on the role of extracellular vesicles is increasing and might be useful to identify new mediators. These vesicles secreted by all types of tissues contain proteins, RNA, and lipids, and are involved in the paracrine or endocrine communications between cells and tissues [30]. For example, it has been shown that exosomes (one category of extracellular vesicles) from lipid-induced insulin-resistant muscles are able to modulate gene expression and proliferation of beta cells in mice [31]. Another study showed that exosomes from adipose tissue macrophages of obese mice were able to induce IR in L6 muscle cells [32]. The miRNA 155 seemed to be implicated in these effects. We cannot rule out that exosomes from adipocytes had a role in our study and could modulate IR and metabolism of skeletal muscle cells.

Some hypotheses could be proposed to explain the regulation of intracellular insulin action and lipid metabolism by LCn-3PUFA in muscle cells. LCn-3PUFA have a strong protective effect against lipotoxicity when they are directly added to muscle cells, allowing their incorporation in cell’s PL. Consequently, the incorporation of LCn-3PUFA in cellular membranes and an improvement in the membrane’s fluidity could facilitate the protein-receptor interactions and, thus, insulin signaling [10]. On the contrary, an excess of saturated FA could induce an elevation of PC content as we observed in moytubes. It remains to be determined if it has a significant impact on insulin sensitivity. 

Our experiments in adipocytes suggested that the co-activation of transcriptional regulators, such as PPARs and LXR by LCn-3PUFA, might also play a role, as we observe a regulation of the mRNA level of several genes (*ADIPOQ, FABP4, CD36*, etc.) that are under the control of these proteins [33,34]. It remains to be determined if similar transcriptional adaptations are involved in the improvement of FA oxidation and detoxification of lipotoxic metabolites in skeletal muscle [35]. The effect of LCn-3PUFA on apelin secretion by adipocytes should be investigated in future studies. Apelin is an adipokine that is known to be insulin-mimetic, which could then modulate the cross-talk between adipose and muscle cells. It was shown to increase glucose utilization in insulin-resistant obese mice [36]. In our study, *APLN* mRNA levels were increased by DPA treatment. Unfortunately, we did not measure its concentration in CM. It was shown that EPA (200 µM) can activate the secretion and gene expression of apelin in the same model of adipocytes [37]. It remains to be determined if such an effect could be observed in situation of IR with a lower and more physiological concentration of LCn-3PUFA. 

In conclusion, DPA has similar effects than EPA on insulin sensitivity in PAL-induced IR in skeletal muscle cells. DPA and EPA partially reversed IR in adipocytes. DPA improved gene expression of key molecules in lipid metabolism and insulin signaling. However, these changes are not sufficient to improve insulin sensitivity of skeletal muscle cells exposed to the CM from insulin-resistant adipocytes. Further studies are necessary to clearly identify which molecules could be responsible for IR transmission from adipocytes to skeletal muscle cells.

## 4. Material and Methods

### 4.1. 3T3-L1 Cell Culture and Treatment.

3T3-L1 cells from ATCC (LGC Standards, Molsheim, France) were cultured in Dulbecco's Modified Eagle's medium (DMEM) (Sigma-Aldrich, Saint-Quentin Fallavier, France) supplemented with 10% calf serum, 100 U/mL penicillin and 100 mg/mL streptomycin in 5% CO_2_ /humidified atmosphere at 37 °C. Two days after confluence (day 0), cells were incubated in DMEM containing 10% fetal bovine serum (Thermo Scientific, Villebon sur Yvette, France), 100 U/mL penicillin, and 100 mg/mL streptomycin (differentiation medium) supplemented with 0.5 mM 3-isobutyl-1-methylxanthine (IBMX), 1 µM dexamethasone, and 10 µg/ml insulin to initiate differentiation into adipocytes. After 48 h, cells were maintained in differentiation medium with insulin only. Insulin was removed after 48 h (day 4) and cells were maintained until day 8 (medium was replaced at day 6). Stock solutions of EPA and DPA (Cayman Chemicals, Montigny-le-bretonneux, France) (50 and 10 mM respectively) were prepared in ethanol (EtOH) and further diluted at 1:1000 in DMEM containing 0.5% of FA-free BSA (ID Bio, Limoges, France). Cells were treated with FA from days 9 to 11. Cells exposed to 0.1% EtOH alone were used as control.

### 4.2. Induction of Insulin Resistance in 3T3-L1 Adipocytes

IR was induced by treating mature adipocytes with 10 µM of insulin (chronic insulin, CI) for the last 16 h of day 10. On day 11, cells were washed twice with Phosphate Buffered Saline (PBS), fatty acid- and insulin-deprived for 6 h before the collection of conditioned media (CM). Once CM were collected, fresh DMEM media was added with 100 nM insulin for 10 min for the analysis of insulin sensitivity.

### 4.3. C2C12 Muscle Cell Culture.

C2C12 myoblasts from ATCC (Molsheim, France) were seeded in 100 mm dishes in proliferation medium composed of DMEM with 4.5 g/L glucose, 2.4 g/L sodium bicarbonate, 10% FBS, and 1% of 100× penicillin and streptomycin mix (100 UI/mL and 100 μg/mL respectively). Cells were kept in a humidified, 37 °C and 5% CO_2_ atmosphere. The medium was changed every 48 h to ensure growth until reaching 80–90% of confluence and the induction of differentiation into myotubes using differentiation medium (2% horse serum instead of 10% FBS in proliferation medium) for five days before cell treatment.

### 4.4. BSA-Bound PAL Solution for Muscle Cell Treatment with Fatty Acids

A solution of 50 mM PAL (Sigma-Aldrich, Saint-Quentin Fallavier, France) in EtOH was prepared and sterilized by filtration before a dilution (1:25) in BSA-enriched DMEM (2% of FA-free BSA) containing 1% penicillin/streptomycin 100×. This 2 mM PAL solution was sonicated 4 min and heated for 15 min at 55 °C.

### 4.5. Fatty Acid Treatment

The BSA-bound PAL solution was diluted four times with BSA-enriched DMEM (0.5 mM final concentration). LCn-3PUFA-supplemented media were prepared by adding stock solution of EPA or DPA (*d* = 1:600) to reach a final concentration of 50 and 10 µM, respectively. All media were kept at 37 °C to ensure BSA-binding before treatment. Differentiated C2C12 muscle cells were washed three times with PBS before exposure to BSA-bound 0.5 mM PAL solution (PAL) with or without 50 µM EPA (PAL+EPA) or 10 µM DPA (PAL + DPA) for 16 h. Control cells were challenged with a FA-free BSA-enriched (2%) DMEM containing 2% of EtOH, without FA. After treatments, cells were stimulated with insulin (100 nM) for 10 min, washed twice with cold PBS, and harvested for protein isolation.

### 4.6. Treatment of Myotubes with Conditioned Media

One volume of CM were first diluted with one volume of fresh DMEM not supplemented with BSA or serum. Then, differentiated C2C12 myotubes were exposed to adipocyte-conditioned media for 16 h before insulin stimulation and harvesting.

### 4.7. Protein Quantification and Western Blotting

C2C12 myotubes and 3T3-L1 adipocytes were harvested in lysis buffer (50 mM HEPES, 150 mM sodium chloride, 10 mM EDTA, 10 mM NaPPi, 25 mM beta-glycerophosphate, 100 mM sodium fluoride, 10% glycerol, 1% sodium orthovanadate, 1% Triton X-100, and 0.5% phosphatase inhibitor cocktail). Protein concentration was determined using BCA protein assay (Thermo Scientific). BSA standard curve and sample preparation and analysis were realized according to the manufacturer’s instructions (Thermo Scientific). Denatured proteins in loading buffer (20 µg) were separated by SDS-PAGE electrophoresis, transferred on PVDF membranes, which were then immunoblotted. Antibody binding was detected using enhanced chemiluminescence (ECL) western blotting substrate (Thermo Scientific) and visualized by Fusion Solo chemiluminescence imaging system (Vilber Lourmat, Marne-la-Vallée, France). Image quantifications were obtained using MultiGauge V3.2 software (Fujifilm, Saint Quentin en Yvelines, France). Phospho ser473/4-(cat. number 9271), total Akt (cat. number 9272) primary antibodies were from Cell Signaling Technology. Glyceraldehyde 3-phosphate dehydrogenase (GAPDH, cat. number G9545) primary antibodies were from Sigma-Aldrich.

### 4.8. Reverse Transcriptase Polymerase Chain Reaction

RT-PCR was performed as previously described [14]. RNA extraction from cells was performed using TRIzol^®^ (Invitrogen, 1 mL/10 cm²) according to the manufacturer's instructions. Chloroform-isoamylalcohol was added (0.2 mL/mL of TRIzol^®^) and samples were mixed and centrifuged 15 min at 12,000× *g* and 4 °C. Aqueous phase containing ribonucleic acid (RNA) was collected, mixed with isopropanol to precipitate RNA and centrifuged (12,000× *g*, 4 °C, 15 min). After centrifugation, the pellet was washed with ethanol 70% (*v*/*v*), dried, and suspended in water. RNA quantification and integrity were evaluated by measuring the ratio of optical density at 260 nm and 280 nm and by agarose gel migration, respectively. Reverse transcription of messenger RNA was performed from 2 µg of total RNA using the High Capacity RNA-to-cDNA Master Mix from Applied Biosystems (Thermo Scientific). A TaqMan low-density array was used for 3T3-L1 adipocyte samples using a 7900HT Fast Real Time PCR system (Applied Biosystems). The entire list of genes investigated and the corresponding relative expression are supplied in the Supplementary Table S1.

### 4.9. Phospholipid Extraction

Lipids were extracted by adding 5 mL chloroform: methanol (1:1, *v*/*v*) to cell extracts. Samples were shaken for 5 min and centrifuged for 10 min at 125× *g*. A total of 2.5 mL of chloroform and 1.5 mL of 0.9% sodium chloride were then added. After shaking for 5 min and centrifugation at 250× *g* for 10 min, the chloroform phase was kept and dried under nitrogen flux. Lipids were suspended in 50 µL of chloroform. Lipid extracts were kept at −20° C until analysis.

### 4.10. Phospholipid Separation and Collection by HPLC

Phospholipid extracts were analyzed in a Thermo Scientific Ultimate 3000 HPLC associated with a charged aerosol detector (Corona, Thermo Scientific) to analyze phospholipid profile and identify the retention time of the different phospholipid classes for collection. Separation was performed using the hydrophilic interaction liquid chromatography (HILIC) method for polar compounds. Briefly, 5–10 µL of phospholipid extracts were injected and separated with an Accucore^TM^ HILIC column (length 1500 mm × diameter 2.1 mm × particle size 2.6 µm, Thermo Scientific). An isocratic inverse phase protocol was used with the following parameters: the mobile phase was (A) acetonitrile/H_2_O (%) 95/5 containing 5 mM ammonium acetate, (B) acetonitrile/H_2_O (%) 50/50; the mobile phase flow and the temperature column were maintained at 0.8 mL/min and 30 °C, respectively. During the run, percentages of A/B were 65/35 at 0 min, 70/30 at 1 min, 85/15 at 20 min, 100/0 at 23 min and 65/35 at 24 min and until the end of the run. Phospholipid collection was performed using an automated fraction collector (Thermo Scientific). Phosphatidylcholine (PC) were collected between 10 min and 12 min and 30 s after injection.

### 4.11. Fatty Acid Profile of PC Fraction

PC fractions were dried under nitrogen and dissolved in boron trifluoride 7% in methanol (Sigma-Aldrich) for FA methylation at 90°C for 2 h. Analysis of fatty acid methyl-esters (FAMEs) was performed on a gas chromatograph (Thermo Electron Corporation, Waltham, MA, USA) coupled with a flame ionization detector using a select FAME (Agilent Technologies, Les Ulis, France) column (0.25 mm inner diameter, 100 m., 0.25 μm film thickness) and helium as the carrier gas (2.6 bar, constant pressure, inlet temperature of 250 °C). Data were expressed as percent of total fatty acids (% TFA) for EPA (20:5n-3) and DPA (22:5n-3). In the cases of pic area below the detection limit, value was replaced by the mean of the corresponding group.

### 4.12. Quantification of Adipokines and Non-Esterified Fatty Acids in Conditioned Media from Mature Adipocytes

IL-6, adiponectin, MCP-1 and CCL5 were quantified using enzyme-linked immunosorbent assay (ELISA) kit from Sigma-aldrich, Assaypro, Thermo Fisher Scientific and R&D Systems, respectively. Non-esterified fatty acids (NEFA) were quantified using a colorimetric kit from Diasys (Grabels, France) according to the manufacturer’s instructions operated on Konelab TM 20 analyzer (Thermo Electron SA, Cergy-Pontoise, France). Regardless of the treatment, every NEFA concentrations found were under the detection limit (10 µM).

### 4.13. Statistical Analyses

All data presented are mean ± standard error about the mean (SEM). For statistical tests, one-way analysis of variance (ANOVA) was performed, followed by a Dunnett post-hoc test taking CI group as reference group, or followed by a Tukey post-hoc test for experiments using palmitic acid on muscle cells and for fatty acid profiles. The number of experiments and samples were indicated in each legend of figures. Analysis have been performed using R software (R Foundation for Statistical Computing, Vienna, Austria), version 3.1.2 and the multcomp package.

## Figures and Tables

**Table 1:** Phosphatidylcholine enrichment in EPA and DPA in cells treated with LCn-3PUFA.

Cell types	3T3-L1				C2C12			
**Treatments**	CTRL	CI	DPA+CI	EPA+CI	CTRL	PAL	PAL+DPA	PAL+EPA
**%DPA**	0.69 ± 0.03^b^	0.84 ± 0.02^b^	2.29 ± 0.54^a^	1.10 ± 0.10^b^	0.48 ± 0.11^b^	n.d.	2.93 ± 0.71^a^	1 ± 0.25^b^
**%EPA**	4.35 ± 1.30^b^	4.74 ± 0.88^b^	10.01 ± 2.5^b^	24.44 ± 2.87^a^	0.94 ± 0.36^b^	0.77 ± 0.18^b^	1.06 ± 0.19^b^	27.13 ± 1.98^a^

Cells were harvested for lipid extraction and isolation of phosphatidylcholine (PC). PC collected were characterized after methylation and analyzed by gas chromatography. They were obtained from 3T3-L1 mature adipocyte after a 48-h treatment with 10 µM docosapentaenoic acid (DPA) or 50 µM eicosapentaenoic acid (EPA) with the last 16 h with or without chronic insulin (CI) or from C2C12 myotubes after 16 h of incubation with 500 µM palmitic acid (PAL) with or without 50 µM EPA or 10 µM DPA. Data are mean ± SEM expressed in % of total fatty acids (*n* = 3–4 obtained after two experiments). In each cell type and line, different letters mean significant differences between groups obtained by ANOVA followed by the Tukey post-hoc test. n.d.: not detected.

## Supplementary Materials

Supplementary materials can be found at http://www.mdpi.com/1422-0067/19/9/2778/s1.

## Abbreviations

AS160	Akt Substrate of 160 kDa
BSA	Bovine Serum Albumin
CM	Conditioned Media
DG	Diacylglycerol
DMEM	Dulbecco’s Modified Eagle Medium
DHA	Docosahexaenoic Acid
DPA	Docosapentaenoic Acid
ECL	Enhanced Chemiluminescence
EPA	Eicosapentaenoic Acid
EtOH	Ethanol
FA	Fatty Acid
FBS	Fetal Bovine Serum
GAPDH	Glyceraldehyde 3-Phosphate Dehydrogenase
HILIC	Hydrophilic Interaction Liquid Chromatography
HPLC	High Performance Liquid Chromatography
HPRT	Hypoxanthine Guanine Phosphoribosyltransferase
IBMX	Isobutyl-Methyl-Xanthine
IL-6	Interleukine-6
IR	Insulin Resistance
IRS	Insulin Receptor Substrate
LCn-3PUFA	Long Chain n-3 Polyunsaturated Fatty Acids
MCP-1	Monocyte Chemoattractant Protein-1
METS	Metabolic Syndrome
PAL	Palmitic acid
PC	Phosphatidyl-Choline
PKC	Protein Kinase C
PL	Phospholipid
PPAR	Peroxisome Proliferator-Activated Receptor
RNA	Ribonucleic Acid
