# Supplementary material for: Modulation of Insulin Resistance and the Adipocyte-Skeletal Muscle Cell Cross-Talk by LCn-3PUFA
Source: Int J Mol Sci. 2018 Sep 15;19(9):2778. doi: 10.3390/ijms19092778 (PMC6164755; doi:10.3390/ijms19092778)
Supplement: Supplementary file 1 [file ijms-19-02778-s001.zip › full table gene list 240818.docx]

**Supplementary table. Effects of CI and LCn-3PUFA treatments on gene expression in 3T3-L1 adipocytes**

| Pathways |  | CTRL | | | | CI | | | DPA + CI | | | EPA + CI | | | ANOVA |
| --- | --- | --- | --- | --- | --- | --- | --- | --- | --- | --- | --- | --- | --- | --- | --- |
| Control of energy metabolism | Adipoq | | 2.12 | ± | 0.47 | 7.60 | ± | 2.31 | 21.12 | ± | 5.15$ | 9.55 | ± | 1.98 | * |
|  | Apln | | 0.69 | ± | 0.15* | 2.25 | ± | 0.29 | 3.42 | ± | 0.34* | 2.70 | ± | 0.23 | *** |
| Fatty acid metabolism | Acadvl | | 0.84 | ± | 0.16 | 1.23 | ± | 0.15 | 2.16 | ± | 0.20* | 1.61 | ± | 0.12 | *** |
|  | Acat1 | | 0.87 | ± | 0.07 | 1.40 | ± | 0.15 | 2.23 | ± | 0.17* | 2.08 | ± | 0.17$ | *** |
|  | Acly | | 0.81 | ± | 0.09 | 0.96 | ± | 0.13 | 1.23 | ± | 0.09 | 1.23 | ± | 0.09 | * |
|  | Cd36 | | 1.52 | ± | 0.48 | 1.63 | ± | 0.17 | 3.21 | ± | 0.37* | 2.79 | ± | 0.24$ | ** |
|  | Cpt1b | | 0.79 | ± | 0.08 | 0.75 | ± | 0.16 | 0.77 | ± | 0.09 | 0.61 | ± | 0.04 | ns |
|  | Fabp4 | | 1.11 | ± | 0.06 | 2.08 | ± | 0.50 | 5.56 | ± | 1.24$ | 4.84 | ± | 0.72 | * |
|  | Hadhb | | 0.90 | ± | 0.13 | 1.05 | ± | 0.17 | 1.56 | ± | 0.11* | 1.36 | ± | 0.11 | ** |
|  | Ppara | | 0.89 | ± | 0.06 | 1.34 | ± | 0.16 | 2.26 | ± | 0.25* | 1.91 | ± | 0.17 | ** |
|  | Ppard | | 0.95 | ± | 0.08 | 1.32 | ± | 0.13 | 1.67 | ± | 0.05 | 1.78 | ± | 0.15$ | ** |
|  | Pparg | | 1.24 | ± | 0.09 | 2.88 | ± | 0.36 | 8.04 | ± | 1.11* | 4.59 | ± | 0.56 | *** |
|  | Slc27a1 | | 1.27 | ± | 0.35 | 1.47 | ± | 0.51 | 1.46 | ± | 0.13 | 1.44 | ± | 0.13 | ns |
| Inflammation & response to stress | Alox5ap | | 0.47 | ± | 0.17 | 0.24 | ± | 0.06 | 0.29 | ± | 0.03 | 0.40 | ± | 0.08 | ns |
|  | Casp1 | | 0.78 | ± | 0.23 | 2.47 | ± | 1.20 | 3.15 | ± | 0.41 | 2.92 | ± | 0.55 | ns |
|  | Ccl2 | | 0.96 | ± | 0.10* | 0.44 | ± | 0.07 | 0.46 | ± | 0.04 | 0.46 | ± | 0.04 | *** |
|  | Ccl5 | | 0.68 | ± | 0.15* | 0.20 | ± | 0.01 | 0.35 | ± | 0.06 | 0.35 | ± | 0.04 | ** |
|  | Ikbkb | | 1.05 | ± | 0.10 | 0.94 | ± | 0.19 | 1.17 | ± | 0.15 | 0.98 | ± | 0.08 | ns |
|  | Il18 | | 0.78 | ± | 0.17 | 1.23 | ± | 0.22 | 1.03 | ± | 0.06 | 1.06 | ± | 0.05 | ns |
|  | Il6 | | 0.81 | ± | 0.11* | 1.72 | ± | 0.28 | 1.28 | ± | 0.07$ | 1.44 | ± | 0.10 | ** |
|  | Nfkb1 | | 0.79 | ± | 0.09 | 0.57 | ± | 0.10 | 0.59 | ± | 0.05 | 0.61 | ± | 0.05 | ns |
|  | Nlrp3 | | 0.67 | ± | 0.21$ | 1.17 | ± | 0.18 | 0.84 | ± | 0.12 | 0.68 | ± | 0.05* | * |
|  | Nos2 | | 0.70 | ± | 0.10 | 0.79 | ± | 0.10 | 0.58 | ± | 0.06 | 0.66 | ± | 0.07 | ns |
|  | Pla2g4a2 | | 0.83 | ± | 0.07 | 0.78 | ± | 0.11 | 0.98 | ± | 0.03 | 1.15 | ± | 0.06* | ** |
|  | Ptgs2 | | 1.50 | ± | 0.77 | 0.60 | ± | 0.10 | 0.88 | ± | 0.13 | 1.19 | ± | 0.19 | ns |
|  | Stat3 | | 1.19 | ± | 0.08 | 1.32 | ± | 0.25 | 1.32 | ± | 0.09 | 1.89 | ± | 0.24 | ns |
|  | Tlr2 | | 0.69 | ± | 0.09 | 0.68 | ± | 0.09 | 0.77 | ± | 0.04 | 0.88 | ± | 0.08 | ns |
|  | Tlr4 | | 0.70 | ± | 0.12 | 0.35 | ± | 0.04 | 0.55 | ± | 0.06 | 0.51 | ± | 0.05 | ns |
|  | Vegfa | | 1.24 | ± | 0.32 | 1.42 | ± | 0.57 | 1.45 | ± | 0.06 | 1.62 | ± | 0.34 | ns |
| Lipid metabolism | Gyk | | 0.74 | ± | 0.08 | 0.76 | ± | 0.10 | 1.05 | ± | 0.08$ | 0.96 | ± | 0.06 | * |
|  | Insig1 | | 0.84 | ± | 0.09 | 1.05 | ± | 0.14 | 1.25 | ± | 0.12 | 0.87 | ± | 0.06 | * |
|  | Irs1 | | 1.18 | ± | 0.15 | 0.91 | ± | 0.07 | 1.44 | ± | 0.11* | 1.24 | ± | 0.10 | p=0.05 |
|  | Irs2 | | 0.92 | ± | 0.06$ | 0.53 | ± | 0.04 | 0.88 | ± | 0.08* | 0.72 | ± | 0.07 | p=0.05 |
|  | Lipe | | 2.00 | ± | 0.64$ | 6.37 | ± | 1.52 | 7.63 | ± | 1.00 | 5.58 | ± | 0.72 | * |
|  | Lpl | | 1.03 | ± | 0.09 | 0.93 | ± | 0.12 | 1.71 | ± | 0.21* | 1.16 | ± | 0.10 | * |
|  | Nr1h3 | | 0.87 | ± | 0.08 | 1.25 | ± | 0.13 | 2.80 | ± | 0.34* | 1.84 | ± | 0.17 | *** |
|  | Plin2 | | 0.88 | ± | 0.09 | 0.90 | ± | 0.20 | 1.28 | ± | 0.11 | 1.33 | ± | 0.09$ | * |
|  | Pnpla2 | | 2.04 | ± | 0.94 | 0.87 | ± | 0.16 | 1.88 | ± | 0.28 | 1.33 | ± | 0.16 | ns |
|  | Srebf2 | | 0.79 | ± | 0.07* | 1.47 | ± | 0.24 | 1.22 | ± | 0.08 | 1.10 | ± | 0.06$ | * |

3T3-L1 preadipocytes were differentiated for 8 days before a 48-hour treatment with 10 µM DPA or 50 µM EPA. Insulin-resistance was induced by adding chronic insulin (10 µM, CI) during the last 16 hours of treatment. Cells were then starved for 6 hours and RNA were extracted to perform qPCR. Relative gene expression of genes related to lipid metabolism (**A**), insulin signaling (**B**), transcription factors (**C**) and regulators of energy metabolism (**D**) are presented. *Nono* gene was used as housekeeping gene. Data are mean ± SEM (n = 5-6 obtained after 3 experiments). The last column presented p values for ANOVA (*p<0.05, **p<0.01 and ***p<0.001 *vs* CI). In other columns, *stand for significant difference (p<0.05) after Dunnett post-hoc test (*vs* CI), $ indicates p<0.1 *vs* CI group and ns means not significant.
