# Supplementary figures and images for: Modulation of Insulin Resistance and the Adipocyte-Skeletal Muscle Cell Cross-Talk by LCn-3PUFA
Source: Int J Mol Sci. 2018 Sep 15;19(9):2778. doi: 10.3390/ijms19092778 (PMC6164755; doi:10.3390/ijms19092778)

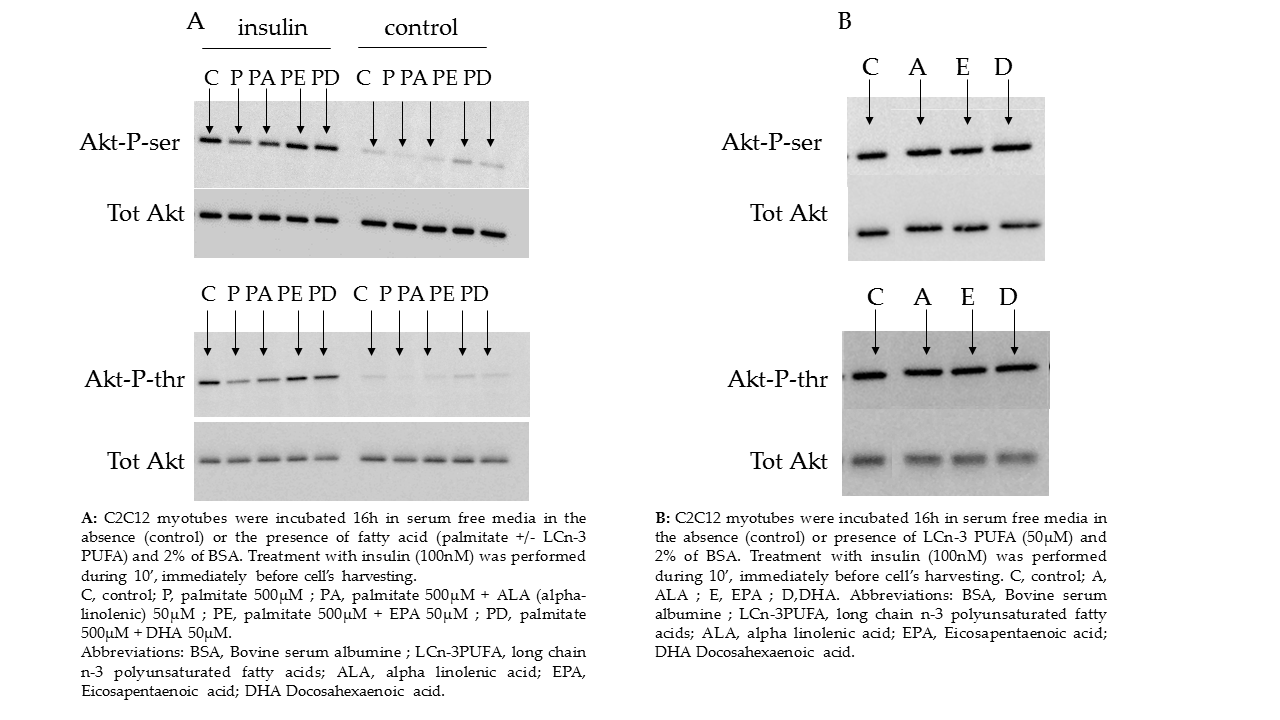

Supplement: Supplementary file 1 [file ijms-19-02778-s001.zip › Supplementary figure 300dpi.tif]
